# Secreted Expression of the Cap Gene of Porcine Circovirus Type 2 in Classical Swine Fever Virus C-Strain: Potential of C-Strain Used as a Vaccine Vector

**DOI:** 10.3390/v9100298

**Published:** 2017-10-16

**Authors:** Lingkai Zhang, Yongfeng Li, Libao Xie, Xiao Wang, Xulei Gao, Yuan Sun, Hua-Ji Qiu

**Affiliations:** 1 State Key Laboratory of Veterinary Biotechnology, Harbin Veterinary Research Institute, Chinese Academy of Agricultural Sciences, Harbin 150069, China; tdzlk1991@163.com (L.Z.); yfli@hvri.ac.cn (Y.L.); xielibao2015@163.com (L.X.); wangxiao2037@126.com (X.W.); sy0604@126.com (Y.S.); 2 Laoshan District Agriculture and Conservancy Bureau, Qingdao 266061, China; gaoxulei2646@163.com

**Keywords:** classical swine fever virus, porcine circovirus type 2, C-strain, live virus vector, bivalent vaccine

## Abstract

Bivalent vaccines based on live attenuated viruses expressing a heterologous protein are an attractive strategy to address co-infections with various pathogens in the field. Considering the excellent efficacy and safety of the lapinized live attenuated vaccine C-strain (HCLV strain) of classical swine fever virus (CSFV), we proposed that C-strain has the potential as a viral vector for developing bivalent vaccines. To this end, we generated three recombinant viruses based on C-strain, one expressing the capsid (*Cap*) gene of porcine circovirus type 2 (PCV2) with the nuclear localization signal (NLS) (rHCLV-2ACap), and the other two expressing the PCV2 *Cap* gene without the NLS yet containing the signal peptide of the prolactin gene (rHCLV-pspCap) or that of the ubiquitin-specific peptidase gene (rHCLV-uspCap). All the recombinant viruses exhibited phenotypes similar to those of the parental virus and produced high-level anti-CSFV neutralizing antibodies (NAbs) in rabbits. Interestingly, rHCLV-uspCap and rHCLV-pspCap, but not rHCLV-2ACap, elicited detectable anti-Cap and -PCV2 NAbs in rabbits. Taken together, our data demonstrate that C-strain can be used as a viral vector to develop bivalent vaccines.

## 1. Introduction

Classical swine fever (CSF) is often a devastating viral disease in pigs, leading to significant economic losses to the pig industry in many countries. The disease is characterized by fever, high mortality, immunosuppression, and reproduction failure in pigs [1]. CSF is caused by classical swine fever virus (CSFV), which belongs to the genus *Pestivirus* of the family *Flaviviridae*, including bovine viral diarrhea virus (BVDV), border disease virus (BDV), and other members. CSFV contains a single-stranded, positive-sense RNA genome, which possesses a 5′-untranslated region (UTR), a single large open reading frame (ORF), and a 3′-UTR. The ORF encodes a precursor polyprotein, upon proteolytic processing, giving rise to four structural proteins (C, E^rns^, E1, and E2) and eight nonstructural proteins (N^pro^, p7, NS2, NS3, NS4A, NS4B, NS5A, and NS5B) [2,3].

Porcine circovirus type 2 (PCV2) has emerged as one of the most devastating viral diseases of pigs. Since the first description at the beginning of the 20th century, PCV2 has been divided into five genotypes, PCV2a, PCV2b, PCV2c, PCV2d, and PCV2e, and the population sizes of the individual genotypes of PCV2 have been changing over time [4,5,6]. PCV2 belongs to the genus *Circovirus* of the family *Circoviridae* and is the primary causative agent of porcine circovirus-associated disease (PCVAD), which results in tremendous economic losses to the swine industry worldwide [7]. The characteristic symptoms of PCVAD are weight loss, enlarged lymph nodes and dyspnea [8]. The genome of PCV2 contains two major ORFs, one coding for the replicase protein and the other coding for the capsid (Cap) protein containing a nuclear localization signal (41 amino acids of the N-terminus) [9]. The Cap protein, as the major immunogenic protein of the virus, can provide complete protection against PCV2 infection [10].

Even though PCV2 infection leads to immune system dysregulation on its own [11], co-infections of PCV2 with other swine pathogens, such as CSFV, porcine reproductive and respiratory syndrome virus (PRRSV), and pseudorabies virus (PRV), usually result in a more severe disease syndrome [12,13]. Bivalent vaccines based on live attenuated viruses expressing the heterologous protein are an attractive strategy to address co-infections. Various bivalent vaccines have been developed [14,15,16,17]. For example, the bivalent vaccine rPRVTJ-delgE/gI/TK-E2 expressing the E2 protein of CSFV in the background of an attenuated PRV was safe and provided complete protection against lethal challenge with PRV and CSFV [16].

C-strain, also called HCLV strain, was developed through hundreds of passages of a highly virulent CSFV in rabbits in China [18,19]. This vaccine strain is an efficacious live attenuated vaccine that is able to protect pigs from lethal challenge of CSFV. Considering the excellent efficacy and safety of C-strain, we proposed that C-strain has the potential as a viral vector for developing bivalent vaccines. To this end, we generated three recombinant viruses based on C-strain expressing the PCV2 capsid protein with or without nuclear localization signal (NLS) and evaluated their immunogenicity in rabbits.

## 2. Materials and Methods

### 2.1. Cells, Viruses, and Vaccine

SK6 (a swine kidney cell line) cells were grown in Dulbecco’s modified Eagle’s medium (DMEM) (Gibco, Gaithersburg, MD, USA) and propagated with 5% fetal bovine serum (FBS) (Gibco) at 37 °C in a humidified 5% CO_2_ incubator. The three recombinant viruses and the parental virus rHCLV (GenBank accession number AY805221) generated from the infectious cDNA clones (below) were cultured in SK6 cells. A commercial inactivated vaccine (LG-vaccine) against PCV2 infections based on the LG strain (GenBank accession number HM038034) was developed by Harbin Veterinary Research Institute of Chinese Academy of Agricultural Sciences. The PCV2 TJ-2013 strain was isolated by our group.

### 2.2. Construction of the Infectious Clones

**Figure 1:**
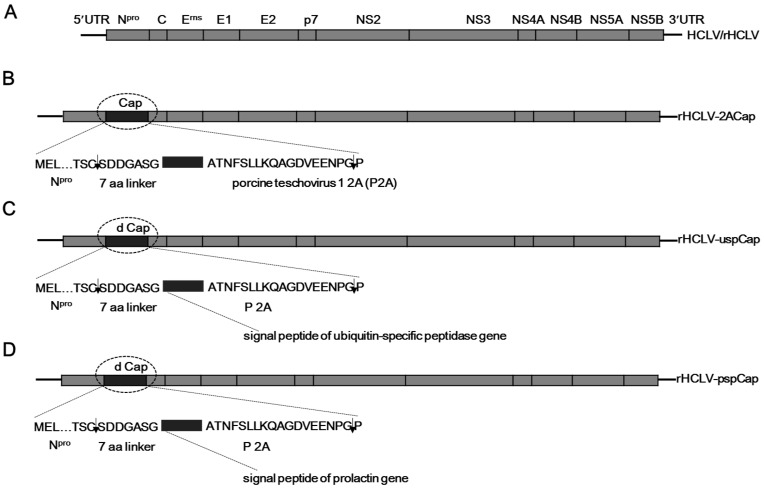
The viral genome organizations of the recombinant viruses derived from the CSFV C-strain. The viral genome organizations of the parental virus HCLV/rHCLV (**A**) and rHCLV-2ACap (**B**). The 7-aa linker and the porcine teschovirus 1 2A (P2A) are shown. The cleavage sites of N^pro^ and P2A are indicated as arrows; (**C**) rHCLV-uspCap harboring the *Cap* gene without the nuclear localization sequence (dCap) is illustrated, and the signal peptide of ubiquitin-specific peptidase gene is inserted in the N-terminus of dCap; (**D**) rHCLV-pspCap harboring dCap is illustrated, and the signal peptide of the prolactin gene is inserted in the N-terminus of dCap.

To construct the infectious clone pHCLV-2ACap (Figure 1), a fusion gene containing a 7-aa linker, the *Cap* gene and the porcine teschovirus 1 2A (P2A) (GenBank accession number NC_003985) self-cleaving peptide encoding sequence were introduced between the *N^pro^* and *C* genes in the infectious full-length cDNA clone of the CSFV C-strain, which was constructed by using the same strategy as the CSFV Shimen strain infectious clone [20]. The other two infectious clones (Figure 1), pHCLV-uspCap containing the signal peptide of the ubiquitin-specific peptidase gene (*usp*) (GenBank accession number ABY84357) of *Lactococcus lactis* and pHCLV-pspCap containing the signal peptide of the bovine prolactin gene (*psp*) (GenBank accession number NP_776378), were constructed. The *N^pro^*, *Cap* or its derivatives, and *C* genes were fused by overlapping PCR. Then the fusion fragment was digested with *Xho* I and *Nsi* I and subsequently ligated into the infectious cDNA clone of C-strain cut with the same enzymes. All primers used for amplifying the fusion genes are listed in Table 1.

### 2.3. Generation of the Recombinant Viruses

The recombinant viruses rHCLV-2ACap, rHCLV-uspCap, and rHCLV-pspCap were generated by transfecting the above infectious clones into SK6 cells as described previously [20]. The transfected cells were passaged 15 times (P0–15) and harvested by three cycles of freezing and thawing. The recombinant viruses were identified using the CSFV antigen test kit (IDEXX, Westbrook, ME, USA) according to the manufacturer’s instructions and tested by an indirect immunofluorescence assay (IFA, see below) using the CSFV E2-specific monoclonal antibody (MAb) HQ06 [21]. In addition, the viral RNA was extracted from the progeny virus supernatants using TRIzol reagent (Invitrogen, Carlsbad, CA, USA) and treated with DNase I to remove the genomic DNA. The isolated RNA was reverse transcribed into cDNA with avian myeloblastosis virus (AMV) reverse transcriptase XL. The *Cap*, *E2*, *NS5B*, *NS5A*, and other genes were amplified from the cDNA by PCR and sequenced. Three samples of each gene were subjected to sequencing by Invitrogen and the results were analyzed by the DNAStar sequence alignment and analysis software (version 7.0) (Madison, WI, USA).

### 2.4. Immunofluorescence Assay and Antigen-Capture ELISA

To examine the expression of the PCV2 Cap protein, SK6 cells were infected with rHCLV-2ACap, rHCLV-uspCap, rHCLV-pspCap or rHCLV in 96-well plates at a multiplicity of infection (MOI) of 0.1. After a 48 h inoculation, the cells were washed three times with phosphate-buffered saline (PBS) and then fixed with cold absolute ethyl alcohol at 4 °C for 15 min. The cells were incubated with a mouse MAb against the PCV2 Cap (1:1000) (Ingenasa, Madrid, Spain) at 37 °C for 2 h, washed three times with PBS, and incubated with an FITC-labeled goat anti-mouse IgG (1:100) (Sigma-Aldrich, St. Louis, MO, USA) at 37 °C for 1 h away from direct light. After washing three times with PBS, the cells were strained with the 4′,6-diamidino-2-phenylindole (DAPI) (1:1000) (Sigma-Aldrich) at room temperature for 10 min and analyzed under a fluorescence microscope (TE2000-U, Nikon, Tokyo, Japan).

SK6 cells were infected with the recombinant viruses. After incubation at 37 °C for 48 h, the PCV2 Cap protein in the supernatants was tested using the PCV2 Cap antigen-capture test kit (Ingenasa) according to the manufacturer’s instructions.

### 2.5. Growth Curves of the Recombinant Viruses

To analyze the growth kinetics of rHCLV-2ACap, rHCLV-uspCap, and rHCLV-pspCap, SK6 cells grown in 24-well plates (Corning, NY, USA) were infected with the recombinant viruses or the parental virus at an MOI of 0.1. After incubation at 37 °C for 2 h, the inocula were replaced with fresh media containing 5% FBS. The supernatants were harvested at 12 h intervals and the titers of each virus were tested by IFA and expressed as 50% tissue culture infective dose (TCID_50_) per milliliter (mL) using the Reed–Münch method [22].

### 2.6. Inoculation and Challenge Experiment in Rabbits

The animal experiments were approved by the Animal Ethics Committee of Harbin Veterinary Research Institute, Chinese Academy of Agricultural Sciences with the license SYXK (Heilongjiang) 2011022 in the February 13, 2017. Thirty-nine 14-week-old New Zealand white rabbits were randomly allocated into seven groups (3 or 6 animals each) and inoculated intravenously with 10^4^ TCID_50_ rHCLV-2ACap (Group A), rHCLV-uspCap (Group B), rHCLV-pspCap (Group C), rHCLV (Group D), C-strain (Group E), 1 mL of LG-vaccine (Group F), or DMEM (Group G), respectively (Table 2). The LG-vaccine group was used as a positive control and the DMEM group as a negative control. After inoculation, the rectal temperatures of all the animals except the LG-vaccine group were recorded every 6 h. At 3 d post-inoculation (dpi), three randomly selected rabbits in each group except the LG-vaccine group were euthanized, and the total CSFV RNA in the spleens of these rabbits was extracted and determined using qRT-PCR, as described previously [23]. The remaining three rabbits were boost-immunized with the corresponding viruses at 3 weeks post-inoculation. At 8 weeks post-inoculation, all the rabbits were euthanized. Serum samples were collected from the rabbits every 1 week following inoculation when the rabbits were kept sedated using a rabbit restraining device.

### 2.7. Blocking Enzyme-Linked Immunosorbent Assay

All serum samples were tested for E2-specific antibodies by a blocking enzyme-linked immunosorbent assay (ELISA)-based CSFV antibody detection kit (IDEXX) and for Cap-specific antibodies by a blocking ELISA-based PCV2 antibody detection kit (Synbiotics, Lyon, France) following the manufacturer’s instructions.

### 2.8. Immunoperoxidase Monolayer Assay (IPMA)

PK-15 cells grown in 96-well plates were infected with PCV2 TJ-2013 strain at an MOI of 0.1. After a 48 h inoculation, the cells were washed three times with PBS and then fixed with cold absolute ethyl alcohol at 4 °C for 15 min. The sera (1:100) of the rabbits and the anti-Cap antibodies (Ingenasa) as a positive control were added to the plates, and incubated for 2 h at 37 °C. After washing three times with PBS, each well was incubated with HRP-conjugated goat anti-rabbit IgG (1:2500) (Sigma-Aldrich) for 1 h. After washing three times with PBS, 3-amino-9-ethylcarbazole (AEC) (Solarbio, Beijing, China) was added to the wells and incubated at room temperature for 10 min, and the cells were washed with PBS and examined using a light microscopy (TE2000-U, Nikon).

### 2.9. Virus Neutralization Assay

Virus neutralization assay was performed as described previously [24]. Briefly, serially 2-fold-diluted sera of the rabbits were incubated with 100 TCID_50_ CSFV Shimen strain or PCV2 TJ-2013 strain at 37 °C. After incubation for 1 h, the mixture was added to PK-15 cells grown in 96-well plate. After incubation for 2 h at 37 °C, the cells were washed with DMEM and cultured in DMEM containing 3% FBS for 48 h. The neutralization activity of the sera in the virus-infected cells was detected using IFA or IPMA.

### 2.10. Statistical Analysis

Differences between groups were examined for statistical significance using Student’s *t*-test by SPSS software (version 14.0) (SPSS Inc., Chicago, IL, USA). An unadjusted *p*-value of less than 0.05 was considered to be significant.

## 3. Results

### 3.1. Generation of the Recombinant Viruses from the Cloned cDNAs

**Figure 2:**
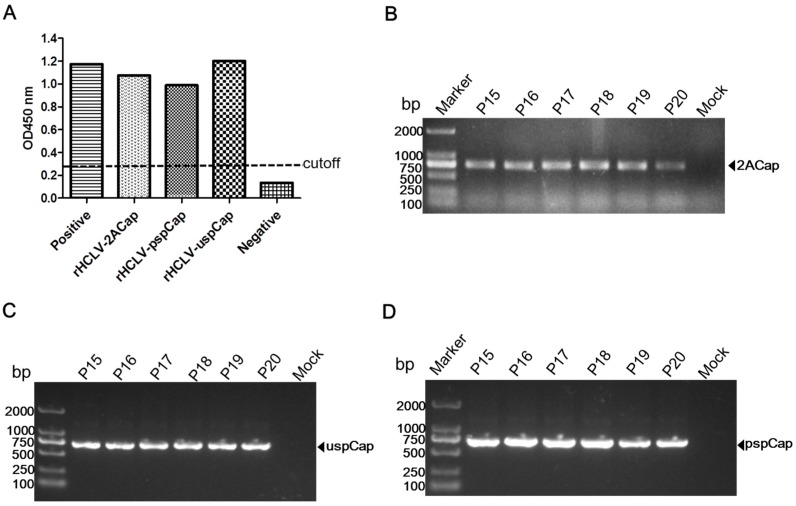
Identification of the generated recombinant viruses. (**A**) The E^rns^ protein of three recombinant viruses was tested by antigen-capture ELISA. The supernatants from the cells inoculated with the recombinant viruses were tested by an antigen-capture ELISA (IDEXX); (**B**–**D**) the inserted genes remained stable in the recombinant viruses. The *Cap* gene was amplified from viral RNA of passage 15 (P15) to P20 rHCLV-2ACap (**B**), rHCLV-uspCap (**C**), and rHCLV-pspCap (**D**) by RT-PCR.

Three recombinant viruses, rHCLV-2ACap, rHCLV-uspCap, and rHCLV-pspCap, were generated from the recombinant infectious cDNA clones. The recombinant viruses were identified by antigen-capture ELISA, RT-PCR, and IFA. The E^rns^ protein of rHCLV-2ACap, rHCLV-uspCap or rHCLV-pspCap of P8–10 was detected by antigen-capture ELISA (Figure 2A). The *Cap*, *E2*, *NS5B*, *NS5A*, and other genes were detected and sequence analysis indicated no mutations in the viral genome. The results demonstrated that the expected recombinant viruses rHCLV-2ACap, rHCLV-uspCap, and rHCLV-pspCap were generated.

### 3.2. Stability of the Inserted Genes of the Recombinant Viruses in SK6 Cells

To investigate the stability of the inserted genes of the recombinant viruses, the viruses were passaged 20 times on SK6 cells. The *Cap* genes of the viruses of P15–20 were detected by RT-PCR (Figure 2B–D). The sequence analysis showed that no mutations occurred in the inserted genes of the recombinant viruses.

### 3.3. Expression of the Cap Protein of the Recombinant Viruses

**Figure 3:**
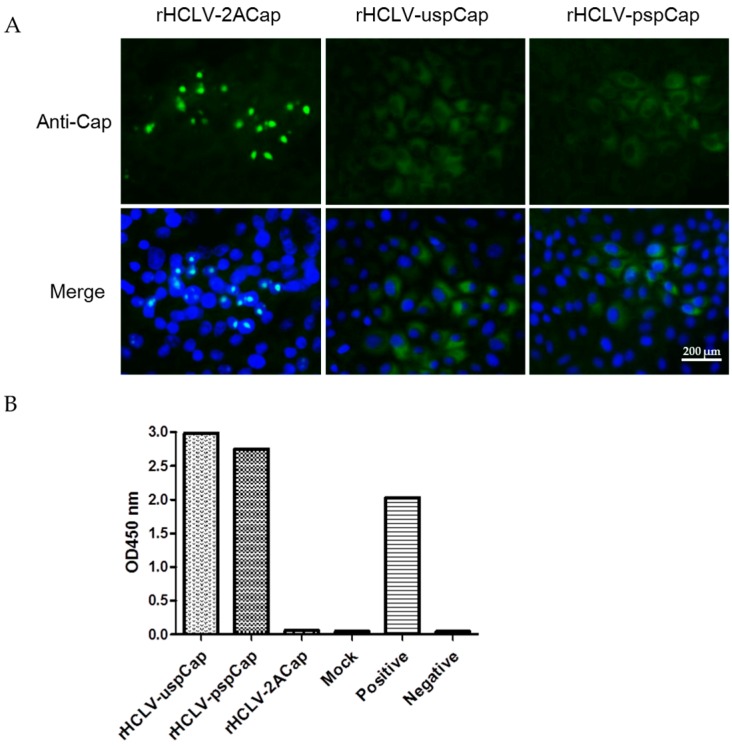
Analysis of the expression of *Cap* gene in the recombinant viruses by IFA (**A**) and by antigen-capture ELISA (**B**). (**A**) SK6 cells were mock treated or infected with the rescued viruses at an MOI of 0.1, respectively. At 48 h post-infection, the cells were processed by indirect immunofluorescence assay using an anti-Cap monoclonal antibody and the nuclei were strained with the 4′,6-diamidino-2-phenylindole (DAPI). The pictures were taken under a fluorescence microscope (TE2000-U, Nikon). The independent experiments were repeated three times. Bar is 200 µm; (**B**) The PCV2 Cap protein in the supernatants from the cells infected with the recombinant viruses or DMEM (Mock control) was tested by an antigen-capture ELISA (Ingenasa).

To determine the expression of the Cap protein of the recombinant viruses, SK6 cells were infected with each recombinant virus and examined at 48 h post-infection. The Cap protein was detected in the nuclei of SK6 cells infected with rHCLV-2ACap, while the Cap protein was detected in the cytoplasm of the cells infected with rHCLV-uspCap or rHCLV-pspCap by IFA (Figure 3A). The antigen-capture ELISA results demonstrated that the Cap protein was secreted into the supernatants of the cells infected with rHCLV-uspCap or rHCLV-pspCap (Figure 3B).

### 3.4. Growth Curves of the Recombinant Viruses

**Figure 4:**
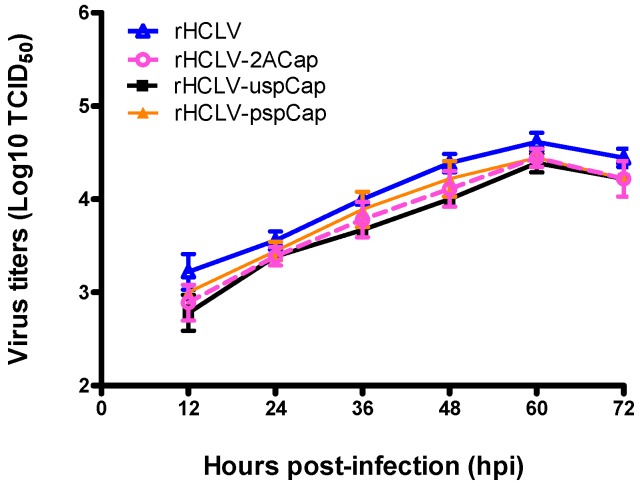
Viral growth properties of the recombinant viruses. SK6 cells were infected with the recombinant viruses or parental virus rHCLV at an MOI of 0.1. The infected cells were harvested by three cycles of freeze-thaw at 12, 24, 36, 48, 60, and 72 h post-inoculation. Viral titers were determined by an indirect immunofluorescence assay and expressed as TCID_50_/mL. The mean value and standard deviation were calculated for three independent experiments.

The growth characteristics of rHCLV-2ACap, rHCLV-uspCap, and rHCLV-pspCap were analyzed relative to the parental virus rHCLV. The growth curves of the recombinant viruses exhibited no significant differences from that of the rHCLV (Figure 4), indicating that the insertion of the *Cap* gene does not affect the viral growth.

### 3.5. The Phenotypes of the Recombinant Viruses in Rabbits

To determine the phenotypes of the recombinant viruses in rabbits, the temperature and viral replication in the spleens of the rabbits inoculated with the viruses were monitored. All of the rabbits inoculated with C-strain or rHCLV developed fever at 36 h post-inoculation, which lasted for 18 to 24 h. Some of the rabbits inoculated with rHCLV-2ACap (4/6), rHCLV-uspCap (4/6), or rHCLV-pspCap (5/6) also exhibited a fever response. At 3 dpi, viral RNA was detected in the spleens of the rabbits inoculated with rHCLV-2ACap, rHCLV-uspCap, or rHCLV-pspCap (Table 3). These data indicate that the recombinants retain the phenotype of the parental virus in rabbits.

### 3.6. Immunogenicity of the Recombinant Viruses in Rabbits

To determine the immunogenicity of the recombinant viruses in rabbits, the anti-E2 and anti-Cap antibodies in the inoculated rabbits were tested by ELISA and serum-virus neutralization test was used to detect the neutralizing antibodies. The antibodies against the CSFV E2 or PCV2 Cap were detectable in rabbits (Table 3). Compared with the DMEM group, high-level anti-CSFV neutralizing antibodies were detected in the rabbits inoculated with rHCLV-2ACap, rHCLV-uspCap, or rHCLV-pspCap (Table 4) from 14 to 35 dpi. Importantly, rHCLV-uspCap and rHCLV-pspCap, but not rHCLV-2ACap, could induce anti-PCV2 neutralizing antibodies at 3 w after boost immunization (Table 5).

## 4. Discussion

To date, a number of PCV2 vaccines have been developed and tested, including inactivated vaccines, DNA vaccines, subunit vaccines, and recombinant chimeric live vaccines [25,26,27]. In addition, several bivalent vaccines containing the *Cap* gene of PCV2 have been reported, including recombinant adenovirus, pseudorabies virus, and swinepox virus [28,29,30]. The advantages of bivalent vaccines include low production costs and less vaccination frequency compared with monovalent vaccines.

CSF is often a highly contagious viral disease causing devastating economic losses and production in the pig industries [31]. C-strain, known as a lapinized live attenuated vaccine strain, has played a vital role in the control of CSF worldwide. The vaccine has been proved to be safe to pigs of all ages and it can provide rapid and complete protection against different genotypes of CSFV [18]. However, the potential use of C-strain as a viral vector was not investigated. In this study, we explored the capacity of C-strain to tolerate the insertion of the *Cap* gene to construct a bivalent vaccine and our data showed the recombinant viruses rHCLV-pspCap and rHCLV-uspCap expressing the secreted Cap protein could induce the antibodies against both CSFV and PCV2, which indicates that C-strain can be used as a viral vector to explore bivalent vaccines.

N^pro^ is an autoprotease unique to pestiviruses that cleaves itself from the polyprotein [32,33]. Using a reverse genetics system, the enhanced green fluorescent protein (EGFP) was expressed separately by the recombinant BVDV depending on the cleavage of N^pro^ and the foot-and-mouth disease virus (FMDV) 2A protein. It was evidenced that the insertion between the *N^pro^* and *C* genes of BVDV did not interfere with the characteristics of the viral vector and foreign protein expression [34]. In addition, another study showed that the porcine teschovirus P2A is shorter and possesses higher cleavage efficiency than the FMDV 2A [35]. In this study, the Cap protein was expressed separately in the recombinant C-strain rHCLV-2ACap, rHCLV-uspCap, or rHCLV-pspCap depending on the self-cleaving N^pro^ and P2A.

Our results demonstrated that the recombinant C-strain rHCLV-2ACap harboring the *Cap* and *P2A* gene exhibited similar phenotypes to the parental virus and produced high-level anti-CSFV antibodies in rabbits. However, no anti-Cap antibodies were produced in the rabbits vaccinated with rHCLV-2ACap. Similar results have been reported by another group. When the green fluorescent protein (*GFP*) gene was inserted in a rinderpest virus (RPV) vaccine strain, no anti-GFP antibodies were produced in the animals vaccinated with the recombinant virus. However, the recombinant virus in which secreted GFP was designed induced an effective antibody response [36]. Therefore, a possible explanation is that the Cap protein of rHCLV-2ACap was translocated to the nuclei due to the NLS of the *Cap* gene. Thus, we designed Cap mutants lacking the NLS for secreted expression in rHCLV-uspCap and rHCLV-pspCap.

Several studies have demonstrated that the ability of producing humoral antibodies by heterologous proteins may be associated with the forms of the protein expression, including intracellularly expressed, secreted forms or on the surface of the cells [36,37]. Possibly, the forms of heterologous protein expression affect the antigen concentration and localization for effectively stimulating the immune system or the efficiency of antigen presentation by the class II major histocompatibility complex [37,38]. The animal immunization experiments showed that the levels of anti-CSFV antibodies showed no significant difference in the rabbits inoculated with rHCLV-2ACap, rHCLV-pspCap, or rHCLV-uspCap. However, compared with rHCLV-2ACap, rHCLV-pspCap, or rHCLV-uspCap expressing the Cap protein without the NLS induced high-level anti-Cap antibodies in rabbits. This implies that the NLS on the N-terminus of the gene may impede the secreted expression of the protein and thus hinder the induction of an immune response in animals.

The efficacy of rHCLV-pspCap and rHCLV-uspCap needs to be further evaluated in pigs. Notably, in the field, pigs are often co-infected by CSFV and other viruses, such as PRRSV and PRV [11]. Since C-strain is an efficacious and safe live attenuated vaccine, it is advantageous to develop a bivalent vaccine based on C-strain to control the co-infections with CSFV and other pathogens.

In conclusion, rHCLV-pspCap and rHCLV-uspCap were able to induce immune responses in rabbits, indicating that C-strain has potential as a viral vector to develop bivalent vaccines.

## Figures and Tables

**Table 1:** Primers used for construction of the recombinant infectious clones.

Primers	Sequences (5′→3′)
pHCLV-2ACap-1F	CACCTCGAGATGCTACGTG
pHCLV-2ACap-1R	CCCACTTGCGCCATCATCGGAGCAACTGGTAACCCACAATGG
pHCLV-2ACap-2F	TCCGATGATGGCGCAAGTGGGACGTATCCAAGGAGGCGTTAC
pHCLV-2ACap-2R	CCACATCGCCGGCCTGTTTCAGCAGCGAAAAGTTTGTGGCAGGGTTAAGTGGGGGTCTTTAAG
pHCLV-2ACap-3F	GCTGAAACAGGCCGGCGATGTGGAGGAGAACCCAGGCCCATCCGATGATGGCGCAAGTGGGAG
pHCLV-2ACap-3R	CTGATGCATGCACCTTGACAGTCGTGAATG
pHCLV-uspCap-1F	CACCTCGAGATGCTACGTGGACGAGGG
pHCLV-uspCap-1R	GGGCTGCAGCAGAAAGTATCACTGTAGACATTAAAATAGCTGAGATAATCTTTTTTTTCATCCCACTTGCGCCATCATC
pHCLV-uspCap-2F	CTTTCTGCTGCAGCCCCGTTGTCAGGTGTTTACGCCCTGGAAATAAGCAGCACCTGCGATGCAGGCATCTTCAACACCC
pHCLV-pspCap-2R	CTGATGCATGCACCTTGACAGTCGTG
pHCLV-pspCap-1F	CACCTCGAGATGCTACGTGGACGAGGG
pHCLV-pspCap-1R	GACCACCAGCAGCAGCAGCAGCCGTGATCCTTTCTGGGAGCTCCCCTTACTGTCCATCCCACTTGCGCCATCATCGGAG
pHCLV-pspCap-2F	GGCTGCTGCTGCTGCTGGTGGTCAGCAACCTGCTGCTGCCTCAGGGGGTGGTCGGAGGCATCTTCAACACCCGCCTCTC
pHCLV-pspCap-2R	CTGATGCATGCACCTTGACAGTCGTG

Note: The sequences coding signal peptides are underlined.

**Table 2:** The animal experiment design.

Group	Prime Immunization	Dose (TCID_50_)	Number	Booster Immunization	Dose (TCID_50_)
A	rHCLV-2ACap	10^4^	6	rHCLV-2ACap	10^4^
B	rHCLV-pspCap	10^4^	6	rHCLV-pspCap	10^4^
C	rHCLV-uspCap	10^4^	6	rHCLV-uspCap	10^4^
D	rHCLV	10^4^	6	rHCLV	10^4^
E	C-strain	10^4^	6	C-strain	10^4^
F	LG-vaccine	1 mL	3	LG-vaccine	1 mL
G	DMEM	1 mL	6	DMEM	1 mL

**Table 3:** Fever response, viral replication, and seroconversion of the rabbits inoculated with the recombinant viruses.

Group	Viruses	Dose (TCID_50_)	Fever Response	Viral Replication	Seroconversion of Antibodies Against CSFV E2 (PCV2 Cap)
A	rHCLV-2ACap	10^4^	4/6	3/3	3/3 (0/3)
B	rHCLV-pspCap	10^4^	5/6	3/3	3/3 (1/3)
C	rHCLV-uspCap	10^4^	4/6	3/3	3/3 (2/3)
D	rHCLV	10^4^	5/6	3/3	3/3 (0/3)
E	C-strain	10^4^	6/6	3/3	3/3 (0/3)
F	LG-vaccine	1 mL	n.d.	n.d.	n.d. (3/3)
G	DMEM	1 mL	0/6	0/3	0/3 (0/3)

Note: n.d., not determined.

**Table 4:** Anti-CSFV neutralizing antibody titers in the sera from the inoculated rabbits.

Groups	Viruses	Days Post-Prime Immunization (Weeks Post-Booster Immunization)					
		0	7	14	21 (0)	28 (1)	35 (2)
A	rHCLV-2ACap	<10	<10	75 ± 32	158 ± 65	710 ± 352	>1028
B	rHCLV-pspCap	<10	<10	89 ± 50	158 ± 95	670 ± 252	>1028
C	rHCLV-uspCap	<10	20	89 ± 50	182 ± 56	630 ± 0	>1028
E	C-strain	<10	21 ± 4	89 ± 50	188 ± 0	710 ± 352	>1028
G	DMEM	<10	<10	<10	<10	<10	<10

**Table 5:** Anti-PCV2 neutralizing antibody titers in the sera from the inoculated rabbits.

Groups	Viruses	Days Post-Prime Immunization (Weeks Post-Booster Immunization)								
		0	7	14	21 (0)	28 (1)	35 (2)	42 (3)	49 (4)	56 (5)
A	rHCLV-2ACap	<10	<10	<10	<10	<10	<10	<10	<10	<10
B	rHCLV-pspCap	<10	<10	<10	<10	<10	<10	80 ± 0	80 ± 0	160 ± 0
C	rHCLV-uspCap	<10	<10	<10	<10	<10	20 ± 0	80 ± 400	160 ± 0	160 ± 0
F	LG-vaccine	<10	<10	<10	<10	<10	40 ± 88	320 ± 0	640 ± 0	640 ± 0
G	DMEM	<10	<10	<10	<10	<10	<10	<10	<10	<10
